# Voir La Vie Sans Rose-Colored Glasses: A Reality Check on Bilingualism and Executive Functioning

**DOI:** 10.1027/1618-3169/a000648

**Published:** 2025-08-18

**Authors:** Kenneth Paap, John Majoubi

**Affiliations:** ^1^ Department of Psychology, San Francisco State University, San Francisco, CA, USA

**Keywords:** executive functioning, attention control, bilingualism, heritability, automaticity

## Abstract

**Abstract:** In a recent article, Ellen Bialystok argued that bilingual experience enhances nonverbal cognition, that its effects are continuous rather than categorical, and that selective attention is the key mechanism underlying cognitive changes in bilinguals. In another recent article, Bialystok argued that bilingual experience modifies cognition by adapting an underlying attention system—one that is limited in resources but becomes more efficient through this adaptation. These claims are critically evaluated drawing on meta-analyses and new empirical tests. These analyses show that any observed advantages are small, inconsistent, and often disappear when accounting for publication bias. A final section describes three key factors that likely explain why bilingualism does not reliably enhance EF. First, dilution and ceiling effects suggest that bilingualism is one of many potential cognitive enhancers (e.g., education, music, mindfulness), making its unique contribution difficult to detect. Second, heritability studies indicate that EF is overwhelmingly genetic in origin, leaving little room for environmental factors such as bilingualism to drive meaningful improvements. Third, automaticity in bilingual language control suggests that proficient bilinguals rely on specialized, task-specific mechanisms rather than domain-general EF, reducing the likelihood of cognitive transfer. Together, these findings challenge the view that bilingualism provides broad cognitive benefits. While bilingualism offers numerous social and linguistic benefits, its impact on nonverbal cognition remains unsubstantiated.

In 2009, the first author (a “word nerd” in his earlier academic career) was retreating from 15 years of university administration, when he wandered into an early Friday morning Bilingualism Session at the Psychonomics conference and was astounded to hear five consecutive talks – Judy Kroll, Tamar Gollan, Ellen Bialystok, Robert Costa, and Viorica Marian – describing how bilingual language control leads to nonlinguistic cognitive advantages. He decided to investigate this exciting phenomena in his new laboratory at San Francisco State University, but failed to generate or replicate any benefits in 15 years of trying (see Paap, 2023). Together with a series of 10 talented student laboratory managers (the penultimate of whom is the second author), we have been swept into what has been described as a “heated controversy” and one of the “most intense debates” in cognitive science. From our perspective, the vigor of the debate should be attributed more to the intrinsic value and interest of the ideas and less to any combative predispositions of the respective proponents on each side of the debate. One aspect of the debate contributing to its endlessness is that the two sides have gravitated to different scientific frameworks: Discovery Science versus Theory-Testing Science.^1^

Briefly, the core mission of Discovery Science (Navarro-Torres et al., 2021) is to focus on open-ended, data-rich exploration of multilingual cognition – especially its variability – without committing prematurely to discrete yes/no hypotheses. It assumes that cognitive outcomes may vary by bilingual experience, language use, context, age, and sociocultural setting and, consequently, that a search for a singular “bilingual advantage” is misguided. Scientific progress in this framework requires mapping the complexity rather than testing oversimplified claims.

Discovery Science proposes that Hypothesis X can be the case. In contrast, the core goal of Theory-Testing Science (Oberauer & Lewandowsky, 2019) proposes that under the conditions specified in the theory, X must be the case. The hypothesis follows deductively from the core assumptions and this tight logical link between theory and hypothesis implies that establishing X as an empirical generalization supports theory T, and conversely, empirically establishing that X is not true counts as evidence against T. This is quintessential theory testing. It offers a chance to obtain strong evidence both in favor and against a theory.^2^ It operates under the auspices of Popper’s (1959) proposition that scientific progress requires theories that are falsifiable and that science can and should be self-correcting.

In their call for more Theory Testing, Oberauer and Lewandowsky assume that scientific progress hinges on testing specific, refutable predictions and that vague or post hoc explanations lead to theoretical stagnation. The Theory-Testing framework encourages controlled experiments, pre-registration, replication, and model comparison and discourages overreliance on exploratory findings and narrative post hoc rationalization. If an effect (e.g., bilingual advantage in EF) cannot be reliably predicted or falsified, it is scientifically weak.

De Bruin et al. (2021) endorse the need to move toward the Theory-Testing framework:

For this field to progress, more specific theories and hypotheses need to be formed regarding the behavioral and neural relationships between bilingualism and executive control…. “The danger with some of these newer frameworks, however, is that they become unprofitably vague…. Differences in executive attention can occur on a wide range of tasks and measures without a clear theory or hypothesis as to when and where these effects should be observed (p. 437).

Rather than choosing one framework over the other, the field may benefit from triangulation and use Discovery Science to identify which bilingual experiences, under what circumstances, with what cognitive demands, might be associated with specified cognitive outcomes. Theory Testing can then be used to rigorously examine these patterns through falsifiable hypotheses and replication.

Having acknowledged the tension between the two frameworks for investigating the relationship between bilingualism and cognition, the next two sections examine two recent updates to Ellen Bialystok’s seminal theory regarding the cognitive consequences of bilingualism. Close attention is paid to precisely what Dr. Bialystok has written, but our focus (and we hope those of our readers) is on the examination of the ideas, not on the person. We believe that one can study how bilingualism interacts with cognition in complex ways without sacrificing falsifiability. But only if one formulates conditional, mechanistic, and contextually grounded hypotheses and one recognizes that complexity does not exempt a theory from being testable.

## Part 1. Bialystok (2025)

With more than 90,000 Google Scholar citations and an h-index of 137 Ellen Bialystok is clearly one of the most productive and influential developmental psychologists in contemporary psychological science. In her recently published reminiscences on a chapter she contributed to the volume by Harris (1992), Bialystok (2025) argues that the following three claims “remain largely intact”:

1. The effects of bilingual experience extend into nonverbal domains.
2. These effects are continuous in nature and not categorical.
3. Selective attention is the key to explaining cognitive change in bilinguals.

Given their influence, it could be argued that our most renown researchers should be held to the highest standards. In that spirit, we beg to differ with the first claim and find the next two difficult to understand. After carefully analyzing these claims and then those made in Bialystok (2024), this review concludes with the consideration of three reasons why bilingual advantages in domain-general attention control are likely to be very small.

### Claim 1. Do the Effects of Bilingual Experience Extend to Nonverbal Domains?

Elaborating on this claim, Bialystok states that “…. Research in my lab and others accumulated evidence supporting the claim that bilinguals out-performed monolinguals on nonverbal cognitive tasks that included conflict….” (Bialystok, 2025, p. 3).

#### Advantages in General Fluid Intelligence (gF)

Recognizing its revolutionary impact, Bialystok (2025) begins the assessment of bilingualism effects on intelligence (gF) with a summary of Peal and Lambert’s (1962) results showing that bilingual 10-year olds outperformed monolinguals on both verbal and nonverbal intelligence tests. The authors observe that “It is not possible to state from the present study whether the more intelligent child became bilingual or whether bilingualism aided his intellectual development….” (Peal & Lambert, 1962, p. 20). Paap (2023) takes this concern for reverse causality one step further by highlighting specific descriptions of their selection criteria that likely favor the assignment of higher gF children to the bilingual group and favor the assignment of lower gF children to the monolingual group. Be that what it may, there is now a rich literature comparing bilinguals to monolinguals in tests of gF.

Bialystok et al. (2022) conducted a pooled analysis of 79 studies (23 children and 56 adult) from the Bialystok laboratory yielding a combined sample of 6,077 participants. There were significant monolingual advantages on verbal measures of gF, but null results on the critical nonverbal measures.

In our work, we have only used adult participants (viz., SFSU students) and nonverbal measures (viz., Raven’s matrices; Raven et al., 1977). Table 1 summarizes the six studies from our laboratory showing the mean Raven’s score (max = 12) for groups of bilinguals and monolinguals and the Bayes Factor calculated for each study. As reported in the published studies, there was never a significant difference between the language groups using an independent groups *t*-test with α = .05 and 1-tailed test. This approach is exploratory (in the spirit of Discovery Science) and should detect true bilingual advantages in spatial gF if they exist for this population of bilinguals. The weighted mean for the 579 bilinguals was 8.53 compared to 8.89 for the 488 monolinguals.

**Table 1:** Bayes Factors for differences in Raven’s scores between bilinguals and monolinguals

Study	*B* mean	*n*_B_	*M* mean	*n*_M_	B–M	BF_01_
Paap and Greenberg (2013)	8.76	49	8.64	55	+.12	6.45
Paap et al. (2017)	8.21	117	8.64	104	−.43	3.42
Paap, Anders-Jefferson, et al. (2018)	8.16	83	9.04	50	−.88	1.27
Paap, Anders-Jefferson, et al. (2019)	8.20	104	8.89	62	−.69	1.32
Mason et al. (2021)	8.86	85	8.85	84	+.01	8.32
Paap, Majoubi, Anders-Jefferson, et al. (2025)	8.99	141	9.18	133	−.17	8.86
*Note*. *B* = bilingual, *M* = monolingual, *n* = sample size, BF_01_ = Bayes Factor (>1 favors null).						

The BFs shown in Table 1 calculate the ratio of the probability of the data given that the null is true to the probability of the data given the alternative. Thus, equal evidence for both hypotheses corresponds to BF = 1.0. Bayes factor analyses, as interpreted by guidelines provided by Jeffreys (1961) or Wetzels et al. (2011), are more conservative than frequentist analyses using standard αs. Note that all six BFs are greater than 1.0 (signaling more evidence for the null than the alternative), despite the fact that the monolingual means are slightly greater than the bilingual means in four of the six studies. Furthermore, note that four of the six studies also show BFs > 3 which, according to the guidelines, indicate substantial evidence for the null hypothesis. In summary, there is very little evidence from the studies of intelligence that the beneficial effects of bilingualism extend to nonverbal domains. One might argue that the evidence favors a null relationship.^3^

#### Advantages in Metalinguistic Ability

The second domain Bialystok examines is metalinguistic ability (i.e., the ability to reflect on or analyze linguistic structure, rather than focusing on the content or meaning alone). Bialystok has done some thought-provoking work on this topic (e.g., Bialystok & Barac, 2012), but as she points out “metalinguistic performance… is related to but not equivalent to cognitive processing, so none of these citations in fact address the central claim” (Bialystok, 2024, p. 2) regarding bilingual effects on nonverbal cognition.

#### Meta-Analytic Tests for Bilingual Advantages in EF

If whimsy strikes, one might propose that the literature reviews (R) appearing in the introduction to empirically based psychology articles have evolved a syntax for citing the relevant literature:

R → Seminal Study + Confirming Study (C_1_ + C_2…_ C_*n*_) but see Inconsistent Study (X_1_ + X_2_), where the Cs and Xs are optional elements.

This syntactic rule lends itself, all too well, to the types of confirmation bias described in Paap, Mason, et al. (2020). In contrast to these ad hoc citations, meta-analyses establish explicit selection criteria, quantitative and standardized measures of effect size, and tools for detecting and correcting publication bias. It is therefore informative, but not particularly surprising, that a series of recent meta-analyses examining the bilingual advantage hypothesis present a remarkably consistent conclusion that is opposite from Bialystok’s first claim (Donnelly et al., 2019; Grundy & Timmer, 2016; Gunnerud et al., 2020; Lehtonen et al., 2018; Lowe et al., 2022; Monnier et al., 2021; Paap, 2019; von Bastian et al., 2017). The consensus is clear: the bilingual advantage, when present, is very small in magnitude. Furthermore, adjustments for publication biases using methods such as PET or PEESE often nullify these effects or even reveal small advantages favoring monolinguals.

An intriguing and recurring pattern across these meta-analyses is the tendency for bilingual advantages to emerge under specific conditions. These include studies characterized by small sample sizes or insufficient control for confounding variables, as well as research conducted by Ellen Bialystok’s laboratory or its affiliates (see Lowe et al. for the most extensive treatment of the study-quality moderator). Contrary to popular narratives, few instances of significant moderation have been found across components of EF, age groups, or types of bilingualism (e.g., early vs. late bilinguals).

#### Meta-Analyses Restricted to Nonverbal Interference Tasks

Nonverbal interference tasks (e.g., Simon, flanker, spatial Stroop) will be treated in this separate section because of the importance Bialystok places on them in tracing the effects of bilingualism from childhood to across the lifespan: “Studying these effects across the lifespan largely replicated the patterns found with children…” (Bialystok, 2025, p. 2). What Bialystok intends to imply is that the bilingual advantages first found with children were replicated with young and older adults. But this has never been the case. Hilchey and Klein (2011) provided the first analytical and conceptual review of the bilingual advantage in cognitive control. They first ask if there is a task-general bilingual inhibitory control advantage (BICA). BICA rests on the assumption that “Frequent use of inhibitory processes involved in language selection in bilinguals will confer general advantages on nonlinguistic interference tasks – that is, those requiring conflict resolution” (p. 628). They conclude that the results are “simply inconsistent with the proposal that bilingualism has a general positive effect on inhibitory control processes” (p. 629). Most of the studies reviewed by Hilchey and Klein involved single measures of EF and small sample sizes. However, within the next 3 years, several large-scale studies reported no bilingual advantages in measures of inhibitory control (Duñabeitia et al., 2014; Gathercole et al., 2014; Paap & Greenberg, 2013). Six years later, the number of tests for bilingual advantages in interference scores had grown to 152 studies. Paap’s (2019) review of these studies clearly show no group differences in children, young adults, or older adults.

#### The Absence of Statistically Significant Monolingual Advantages

In a meta-analysis that comes to strikingly different conclusions, Grundy (2020) raises a number of important issues, but they do not change the meta-analytic “facts” which are, that the mean effect sizes are very small and not distinguishable from zero when corrected for publication bias. Grundy’s main counter-argument is that the number of significant bilingual advantages is far larger than the number of the significant monolingual advantages. Grundy rules out the role of publication bias because the 37 studies that were not published in journals were just as likely to find bilinguals outperforming monolinguals as the published studies. Although Bayes Factors were used to formally test the hypothesis that the number of significant positive results was greater than the number of negative, the use of Bayes Factors does not mitigate the possibility that there is a substantial bias in the use of Questionable Research Practices (QRPs) to induce small, but statistically significant, positive findings.

Paap (2023) discusses the potential role of QRPs and the twin devil of confirmation bias at length. These biases are hidden from readers because they involve decisions about what to report. They are probably more prevalent than most psychologists suspect. In a survey of 2,000 research psychologists, John et al. (2012) reported that 48% have submitted only the experiments that “worked” and that 65% admitted that they failed to report all the dependent variables measured and analyzed. Other prevalent practices from the John et al. survey include deciding to collect more data when the results are not significant (57%) and rerunning analyses with outliers removed (41%).

In a simulation, Bakker et al. (2012) showed that applying these QRPs to simulated results that initially produced a null outcome can cause the rate of false positives to jump to nearly .40. It seems plausible that many researchers squeeze one small, but significant, bilingual advantage from their data but have no incentive to coax a numerical monolingual advantage to significance. In fact, there is more likely to be a disincentive to produce a significant result in what the field believes to be in the wrong direction. This is likely to be the cause of the disparity between significant bilingual advantages and bilingual disadvantages. Given Grundy’s observation that significant bilingual advantages occur more often than significant monolingual advantages in both the published research and in unpublished papers (e.g., dissertations, master’s theses, and conference posters), it appears that pressure or bias against significant results, but unexpected negative results, is comparable both among published academics and among budding academics hoping to generate expected results and to impress their mentors. Perhaps publication bias is too narrow a term to refer to the power of confirmation bias as described by Paap, Mason, et al. (2020).

### Claim 2. Effects of Bilingualism Are Continuous, Not Categorical

Bialystok (1992) surmised that the effects of bilingual experience not only extend into nonverbal domains but also that the effects are continuous in nature and not categorical. What Bialystok and other proponents mean by this (and what the implications are) is not always clear. The primary consequence of this observation for Bialystok (2025) appears to be for the design and analysis of studies testing for the effects of bilingualism on brain and cognition. She states that “In the majority of early research, and probably still the majority of research conducted currently, the empirical design was based on group comparisons” (p. 3). Is testing for bilingual effects using between-group comparisons always the wrong thing to do?

#### The More Difficult Problem of Multidimensionality

An undeniable problem with simple between-group designs is that bilingual experience consists of many different dimensions, and we do not know which facets (if any) are causally related to putative increases in nonverbal cognition. For ease of exposition, the term “active ingredient” will be used for a hypothetical dimension of bilingual experience that does enhance nonverbal cognition. For purposes of illustration, Bialystok (2025) poses the hypothetical and simplified case that frequency-of-use (some measure of the degree to which there is balanced used of both languages) is an active ingredient, but knowledge-of-L2 (some measure of proficiency) is not. In this scenario, “the cognitive outcomes for groups described as monolingual and bilingual depend on which criterion of bilingualism was used to create the groups” (Bialystok, 2025, p. 3). This is clearly a problem, but it is not caused by treating bilingualism as a categorical variable, it is a problem caused by treating bilingual experience as unidimensional. Note that the same problem of arriving at two opposing conclusions would still occur if bilingual experience and frequency-of-use were measured continuously and used as predictors in a regression analysis of nonverbal cognitive performance. Given the presumptions of this hypothetical case, frequency-of-use would be a significant predictor while degree of proficiency would not – the same discordant pattern as obtained in the two groups analysis. We can probably all agree that bilingual experience should be treated as multidimensional as we sift and winnow our way to discovering any active ingredients.

#### The Value of Also Using Language-Group Analyses

Any one test of the hypothesis that bilingualism affects nonverbal cognition must select one or more facets of bilingual experience, measure them categorically or continuously, and select one or more measures of nonverbal cognition. Starting with our first published study on this topic (Paap & Greenberg, 2013), we have exercised the strategy of always analyzing the data both as a between-group comparison and using regression. This seemed necessary because almost all our tests have yielded statistically null results^4^ and we wanted to demonstrate that there were no bilingualism effects hidden behind the choice of tests. Since Paap et al. (2014), we also started to report Bayes Factors (BF) for each test and always observed more evidence favoring the null hypothesis than the alternative. In the majority of cases, the evidence favoring the null was “substantial” using Jeffreys’ (1961) guidelines.^5^

A potential disadvantage of testing for differences between monolinguals and bilinguals is that the sample distribution of a bilingualism measure may have many participants in the middle who are neither purely monolinguals nor model bilinguals. Like many others, when doing an analysis between groups, we exclude participants who are ambiguous. For example, some SFSU students claim very low proficiency in any language other than English, but report using this other language quite often. Conversely, some of our students are highly fluent in a non-English language but report that they currently use it less than 10% of the time. Participants like these are assigned to the “ambiguous” category and deleted from the tests comparing bilinguals to monolinguals. The goal is to compare unambiguously pure monolinguals to bilinguals who are proficient and use both languages. Potentially these tests of “extreme” groups can sometimes be more sensitive than a regression analysis on a sample that includes a fair number of participants in the ambiguous center of the bilingualism distribution where any positive effects of bilingualism might be modest.

Although data can be fit to any function, most tests that treat bilingualism as a continuous measure use linear tests. Bialystok (2025) justifies this by citing research that shows a linear relation between aspects of bilingual experience and aspects of structural and functional changes in the brain. This might be a bit selective or over-simplified as other researchers emphasize nonlinear relationships (e.g., Pliatsikas’ dynamic structuring model, 2020 or Gallo et al.’s inverted U-shaped relationship for gray-matter volume in the caudate nucleus as a function of bilingualism, 2024).

#### The Superiority of Continuous Measures Is Nuanced

Is it foreordained that causal effects are more likely to be continuous than categorical? Of course not. The A allele results, categorically, in an A blood type. Categorical outcomes can occur even when the underlying cause is a continuous variable. For example, warmer incubation temperatures typically produce more female sea turtles and fewer males (Morreale et al., 1982). Closer to home, bilingual individuals often develop a categorical preference for when and how they switch between languages. For example, speaking a heritage language with family and the community language elsewhere. Some bilinguals show categorical activation in different brain regions for processing L1 versus L2 (Kim et al., 1997).

In a different vein, many computational models of cognition assume that continuous activation values in individual nodes or networks are governed by thresholds (e.g., McClelland & Rumelhart’s influential interactive activation model, 1981). The final conversion from continuous amounts of activation (or evidence or utility) to a categorical response (e.g., Luce’s Choice Rule, 1959) is a foundational principle in decision-making and mathematical psychology. It may be the case that a continuous measure of some facet of bilingualism is a superior analytic tool for some specific hypothesis about the effects of bilingualism on brain or behavior, but it is not given and is likely to rely on a detailed and nuanced argument that is heavily context dependent.

### Claim 3. Is Selective Attention the Key to Explaining Cognitive Change in Bilinguals?

Bialystok’s (2025) third claim is that selective attention is the key to explaining bilingual advantages in nonverbal cognition. She traces this back to her chapter (Bialystok, 1992) in the Harris (1992) volume where she observed that “…there was no difference between monolingual and bilingual children in their ability to encode and access explicit representations” but “that bilingualism enhances children’s ability to control attention in the context of distraction, an ability that I referred to as the selective attention advantage for bilingual children” (p. 3).

#### The Now Forgotten Swerve to Inhibitory Control

Bialystok’s reflection on her 1992 chapter might leave the impression that she has maintained her focus on selective attention since the 90s. However, both Klein (2015) and Paap (2023) have selected related (but different) quotes from her landmark 2001 book to support the view that in 2001, Bialystok attributed the putative bilingual advantages to inhibition rather than selective attention. “Tasks that showed a bilingual advantage had in common a misleading context and moderate conceptual demands . . . what bilingual children are able to do is to inhibit attention to misleading information of greater salience or complexity than monolingual children can” (pp. 213–214).“This inhibition is undoubtedly achieved by means of processes carried out in the frontal lobe. If this model is correct, then bilingual children experience extensive practice of these functions in the first few years of life, at least once both languages are known to a sufficient level of proficiency to offer viable processing systems. It would appear that this practice in inhibiting linguistic processing carries over to processing in highly disparate domains” (p. 216). At this time, Bialystok has clearly placed a bet on the importance of inhibition: “…what bilingual children are able to do is to inhibit attention to misleading information of greater salience or complexity than monolingual children do … Inhibition is the essential factor in distinguishing the performance of the bilingual children, so it may be that bilingualism exerts its effect selectively on the inhibition component of attention” (p. 214).

#### The Role of Miyake’s Model of EF in the Bilingual-Advantage Debate

Despite her endorsement of an inhibitory-control account, Bialystok (2025) argues that the popularity of selective-attention account was derailed as the field became enamored with the influential model of EF introduced by Miyake et al. (2000). This section of Bialystok (2025) will strike many readers as somewhat incoherent. Minimally some misconceptions are propagated. Bialystok starts on firm ground, noting that Miyake et al. (2000) proposed that EF “…consisted of three separable but related processes – shifting, updating, and inhibition” (p. 4). But a small misstep takes Bialystok down a primrose path when she states that: “the model was tested using a set of specific tasks, such as Stroop, switching, and antisaccade. Each of which was designated as an index for one of the components” (p. 4). This is not literally correct and can easily be misread as asserting that a measure from one task (e.g., flanker) was used to estimate one component of EF (e.g., inhibition). Rather, Miyake et al. used three different tasks to extract a latent variable for each of the three components because each component of EF was assumed to be a domain-general component. Indeed, for each component, the three separate tasks loaded on a coherent latent variable.

Bialystok asserts that “As a resource, selective attention is different from the EF framework because it does not predict bilingual advantages on the basis of type of task (inhibition, working memory, etc.) but rather by the extent to which the task requires attention control. Unlike the three components of the EF framework, attention is an underlying resource that varies along a quantitative continuum” (p. 4). This misrepresents the conceptual assumptions underlying the model developed by Miyake and colleagues. The Miyake et al. (2000) model is no different from Bialystok and Craik’s (2022) Attention Control^6^ (AC) model in assuming a domain-general resource that affects performance on any difficult task that requires top-down cognitive control. Inhibition requires effort to suppress prepotent responses or to resist interference. Shifting requires cognitive flexibility to disengage from one mental set and adopt a new one, which also demands attentional resources. Updating involves maintaining and manipulating information in working memory which is resource-intensive. These components are thought to operate across a wide variety of tasks and contexts rather than being tied to specific cognitive domains such as language, math, or spatial processing. The domain-general nature of these components reflects their flexibility in supporting different kinds of goal-directed behaviors. The difference is that Miyake et al. (2000) model has three separable but related components whereas Bialystok and Craik’s AC model is unitary. It is worth repeating that Bialystok is simply wrong when she asserts: “Executive function is specific and task-based whereas selective attention refers to a general resource” (p. 4). The meta-analyses showing no evidence for bilingual advantages in nonverbal cognition cannot be dismissed by calling them tests of task-specific EF. It is telling that the examples of tasks that are affected by AC provided by Bialystok and Craik (2022) include the most classic tests of the inhibition component of EF: Stroop, flanker, and Simon. It seems that bilingual advantages on these traditional nonverbal interference tasks are treated as clear evidence that bilingualism enhances domain-general EF whenever significant advantages occur and are dismissed as being too easy whenever they yield null results.

#### The Role of Latent-Variable Analyses in Theory Testing

Bialystok’s (1992) early proposal that bilinguals enjoy an advantage in selective attention eventually evolved into the theory of AC presented in Bialystok and Craik (2022) and updated in Bialystok (2024). As argued in the preceding section, both Miyake et al.’s (2000) theory of EF and the AC theory assume that goal-directed and top-down control recruits domain-general processing resources. The theories differ in that the former assumes three separable but related components whereas the latter assume a single resource. This distinction has narrowed because Miyake and Friedman (2012) now favor a model that includes a common EF.

To review, the 2000 model showed that a general EF ability can be modeled as consisting of three latent variables (the updating, switching, and inhibitory components) that are each recruited to enable performance in a variety of different tasks. Miyake and Friedman (2012) compared the fit of this model to a more complex second-order (aka nested) model where the nine observed measures are allowed to load on common EF, and the three latent variables compete in accounting for the remaining variance. The best solution for the second-order model resulted in all nine measures loading on the common EF and with only two of the nested components (updating and switching) still making unique contributions. Putting this together, the model supports a theory of a general EF ability with separate updating and switching components and an inhibition component that is not separable, but moderately linked to general EF ability. This analysis led Miyake and Friedman (2012) to conclude that EF has both unity (a common EF) and diversity (additional specific abilities that are not task-specific but are associated with processes involved in switching and updating). Thus, both theories now embrace a domain-general resource (unity), but the unity and diversity theory includes an additional pair of separable components.

One needs to consider not only what a theory assumes but also the degree to which the hypothesized structure and functions are confirmed by behavioral testing. In this regard, the Miyake et al. (2000) model has encountered some controversy. For example, a study by Rey-Mermet et al. (2019) used six tasks assumed to reflect “Inhibition of Prepotent Responses” and five assumed to reflect “Resistance to Distraction.” The former assumes that inhibition is applied to the preparation or execution of a response. For example, the Go/No Go task that requires participants to not respond on some trials when a “No Go” signal is presented should load on this factor. The latter, “Resistance to Distraction,” is required in the flanker task to filter out the flanking arrows on an incongruent trial. Bayesian hypothesis testing showed that the data provide ambiguous evidence as to whether there is one inhibition factor or two. Furthermore, latent variables for inhibition tend to be dominated by a single task (viz., the antisaccade task). Rey-Mermet et al. concluded that nonverbal tests used to assess “inhibition” do not measure a common, underlying construct but instead measure the highly task-specific ability to resolve the interference arising in each task. For them, the “... inevitable implication is that studies using a single laboratory paradigm for assessing or investigating inhibition do not warrant generalization beyond the specific paradigm studied” (p. 515). We agree that tests of the effects of bilingualism on inhibition (or any EF component or on a unitary AC mechanism) should use latent variables and avoid single tasks.

Given Rey-Mermet et al.’s study, it is somewhat surprising that in the Miyake et al. (2000) study, measures derived from the antisaccade, stop-signal, and Stroop task produced a coherent latent variable for Inhibition. Furthermore, in this case, the three-factor model fit the data significantly better than a one-factor model.

#### Evidence for Unidimensional Attention Control

The previous section describes a fair amount of latent-variable evidence for a unity and diversity model of EF with three components. How does this compare to the evidence for a unidimensional AC? Bialystok has provided no evidence for single-factor theory of AC. Although Bialystok and Craik’s “attention control” seems to have been borrowed and adapted from Engle’s construct with the same name, there are disconcerting disconnects between the two. Engle has invested heavily in the need to develop a set of measures for AC and has identified sets of tasks (e.g., antisaccade, flanker DL, visual arrays in Draheim et al., 2020 and the Square tasks in Burgoyne et al., 2023) that form coherent latent variables. Furthermore, Engle’s latent variable for AC impressively mediates the relationship between a latent variable for WMC and a latent variable for intelligence (Draheim et al., 2020). A latent variable for AC also predicted 75% of the variance in a complex real-world task (Burgoyne et al., 2023). In contrast, Bialystok and Craik (2022) offer a laundry list (see their Table 2) of studies “for which better bilingual performance is more plausibly attributed to attention control than to inhibition” but no plan or method for identifying a set of measures that are truly unidimensional.

Although AC is described as a continuous and unidimensional ability (not one composed of discrete and separable components such as the inhibition, shifting, and updating), Bialystok does distinguish between different aspects^7^ of AC. A surprising amount of research capital was spent on testing the general hypothesis that bilinguals, compared to monolinguals, were better at engaging or disengaging attention. Several tasks initially showed results consistent with the view that bilinguals were quicker to engage or disengage attention, but these positive findings proved difficult to replicate.

##### Congruency Sequence Effects

Grundy et al. (2017) pursued the hypothesis that bilinguals are better than monolinguals at disengaging attention by comparing the magnitude of congruency sequence effects (CSEs). The underlying logic is somewhat convoluted. CSEs are robust context effects observed in many choice RT tasks that include both congruent and incongruent trials. CSEs are defined as smaller interference effects when the previous trial is incongruent compared to when it is congruent. The logic of Grundy et al.’s prediction starts with the hypothesis “that bilinguals can more rapidly disengage attention from irrelevant information than monolinguals in a simple flanker task” (p. 42). On an incongruent trial of a flanker task, the central target arrow might point to the right while the irrelevant flankers point to the left. Other factors equal, individuals who can quickly disengage from the incongruent flankers and focus attention on the center location will respond faster on these incongruent trials and have smaller interference scores than those who focus attention more slowly or broadly. Given the preponderance of null results in nonverbal interference tasks (see discussion of Claim 1 above), Grundy et al. concedes that bilinguals do not have smaller flanker effects compared to monolinguals. Surprisingly (at least to us), the hypothesis that bilinguals can more rapidly disengage attention from irrelevant information (e.g., the irrelevant flankers in the flanker task) was repurposed to whether they can more rapidly disengage from the cognitive control required on trial *n* − 1 (e.g., an incongruent trial) to a plan suited to the current trial n (e.g., a congruent trial). In other words, the Grundy argument is not concerned with disengaging attention from an external stimulus in a specific spatial location, but rather disengaging (switching) from one internal control-plan to a different one. This is quite a leap and strikes us more like a metaphor than as an explanation for CSEs. In any event, let us consider Grundy’s account of CSE’s and whether the magnitude of the CSE can be used as an index of the disengagement of attention.

Assume that attention control is exercised within a single trial when the detection of conflict between the incongruent flankers and target triggers an upregulation of the relevant information and/or the inhibition of the irrelevant information. Thus, the attentional control triggered and implemented on trial n is not intentional preparation for trial *n* + 1, but an unintended carryover effect from trial *n*. If the trial type happens to repeat (e.g., iI or cC), the carryover will benefit the decision on trial *n* + 1, but it will produce a cost if the trial type switches (e.g., cI or iC). In this scenario, disengagement would involve shifting away from the control plan most recently used and into a neutral state that could be rapidly adapted to the type of trial (congruent or incongruent) that occurs next. Rapid disengagers would be in a neutral state more often than slow disengagers and would therefore have smaller SCEs.

In their first two experiments using a flanker task, Grundy et al. (2017) observed no language-group differences in the magnitude of the simple flanker effect (incongruent trial RT minus congruent trial RT), but bilinguals did have significantly smaller CSEs compared to monolinguals. The smaller CSE was interpreted as reflecting “.... more rapid disengagement of attention and greater ability to refocus on the current trial” (p. 45; Grundy et al., 2017). The findings are asserted to “…provide insights into why some studies show bilingual advantages on executive control tasks and some do not” p. 52. “…bilinguals were more efficient than monolinguals at disengaging attention from the previous trial in order to devote resources to the current trial” (p. 330*,* Bialystok & Grundy, 2018).

Shortly thereafter, Paap, Myuz, et al. (2019) published a systematic review on the “bilingual advantage” in CSE magnitude that showed 10 failures to replicate. Half of these tests used the flanker task (like Grundy, Chung-Fat-Yim, et al., 2017) and half used the Simon task. The sample sizes were greater than those used by Grundy, and none of the tests produced significant language-group differences in the standard interference score, global RT, or the magnitude of the CSEs. The nonsignificant numerical trends showed smaller CSEs for the monolinguals, not the bilinguals, in eight of the 10 tests.

Two newer studies were not included in the Paap (2019) review. Goldsmith et al. (2023) recruited 492 children in the age range of 9 to 14 years from Canada, China, and Lebanon. From the original sample, 105 bilinguals were matched to 105 monolinguals of the same exact age, same exact digit span, and same SES (based on a composite of family income, parental employment status, and parental education). A size-congruency task was used in which participants had to respond to the numerically larger digit while ignoring its physical size. This task is arguably preferable for measuring the CSE as it avoids possible confounding effects of stimulus repetition and contingency learning (for discussion, see Paap, Myuz, et al., 2019). In the analysis of RTs, there were no statistically significant differences between the language groups in global RT, the classical congruency effect, or the CSEs.

The Grundy et al. article is highly cited as providing evidence that bilinguals are better at disengaging attention, but its persuasiveness collapses under the weight (a) of failures to replicate the finding that bilinguals had smaller CSEs, (b) of alternative explanations for congruency sequence effects that have nothing to do with cognitive control, and (c) the fact that these effects are not adaptive in the sense of leading to better performance.^8^

##### Conjunctive Visual Search

Friesen et al. (2015) reported a bilingual advantage in conjunctive visual search. In this task, participants must search through a visual display for a target (viz. a green triangle) and press the “1” key if it was present and the “0” key if it was not. A target, if presented, randomly appeared in one of 26 locations and was accompanied by either 0, 5, 15, or 25 distractors that were either purple triangles or blue diamonds. Identifying the target (a green triangle) requires conjoining the features (i.e., considering both color and shape) because there are both green distractors and triangle distractors. Bilinguals outperformed monolinguals in this study.

Friesen et al. infer that “faster target identification indicates that bilinguals were better at engaging and disengaging selective attention to identify targets in the display” (p. 8). The underlying logic of this conclusion is not explicit but must include the following steps: (1) the control mechanisms for engagement and disengagement are domain-general and (2) these mechanisms strengthen with use. Why mere use should lead to increased domain-general ability is rarely discussed. A well-known exception is Baumeister’s strength model. The model assumes that most tasks require general processing resources and that the depletion of general processing resources lead to an increase in total available resources (Baumeister & Vohs, 2016).

Although Ratiu et al. (2017) tested the same research question as Friesen et al. (2015), they failed to find any bilingual advantages in search time across a series of three experiments that used eye movements to separate search time from decision time. The only reliable group difference was observed in Experiment 3 and that was a bilingual disadvantage in decision times. Ratiu et al. (2017) conclude that their results show no bilingual advantages in attention, response initiation, or overall search performance.

The materials used by Ratiu et al. were quite different from Friesen et al.’s. Thus, Paap, Anders-Jefferson, et al. (2018) conducted a close replication of the critical conditions of the Friesen et al. (2015) search task. Their results also showed no differences between the language groups. The best dependent measure is the slope of the best fitting straight line for search time as a function of the number of distractors. For both target-present and target-absent slope, those group means differed in slope by less than 2 milliseconds.

##### Morphing Ambiguous Objects

Another task advanced as reflecting the ability to disengage attention used a series of line drawings that gradually morphed from the starting object to a different object. Chung-Fat-Yim et al. (2017) reported a bilingual advantage in this ambiguous figures task. They concluded that bilinguals were better able to disengage attention from the salient features consistent with the first object to selectively attend to features consistent with the second object. In a close replication and using a larger sample size, Paap, Anders-Jefferson, et al. (2018) found no significant differences between monolinguals and bilinguals. However, two earlier studies using children, rather than young adults, had also shown a bilingual advantage in a task using ambiguous figures (Bialystok & Shapero, 2005; Wimmer & Marx, 2014).

##### Inhibition of Return (IOR)

IOR is a well-investigated phenomenon in cognitive psychology showing that when a peripheral cue elicits a covert shift of attention, there is an initial period where detection of a new stimulus at the cued location is facilitated, to a phase when detection at that location is actually delayed (Klein, 2000). The latter interval is referred to as IOR and its onset may reflect how quickly an individual shifts from an engagement phase to a disengagement phase. Grundy et al. (2017) cited Mishra et al.’s (2012) report that high-proficiency bilinguals display inhibition-of-return (IOR) effects at earlier cue–target onset asynchronies (CTOAs) than low-proficiency bilinguals as support for the disengagement hypothesis. Mishra’s study did not include monolinguals and, consequently, does not directly test a bilingual advantage hypothesis. In a study (Hernández et al., 2010) that actually did compare bilinguals to monolinguals, there were no group differences in the time course of IOR. Furthermore, in a replication and extension of their earlier work, Saint-Aubin et al. (2018) tested a large sample of English–French bilinguals and reported no effects of L2 proficiency on IOR. Saint-Aubin et al. concluded that there is no reliable evidence that mastering a second language leads to faster or more potent disengagement of attention.

More recently, Grundy et al. (2020) revived this line of evidence using the typical IOR paradigm and concurrent EEG recordings. Four different measures of bilingualism were solicited (measured as L2 proficiency, age of acquisition, usage, and switching frequency) in tests between groups based on a median split or as continuous predictor variables using regression. Only one dimension of bilingualism, L2 proficiency, significantly predicted the magnitude of IOR and only in the regression analyses. But recall that Saint-Aubin et al. also used L2 proficiency as a continuous predictor and, furthermore, did three separate analyses based on self-ratings, reading comprehension, and reading rate. All three produced null results. The Saint-Aubin et al. tests were also more powerful as they used 100 participants compared to only 44. This does not mean that Grundy et al.’s results should be discounted, but one can confidently assert that they are not robust.

##### Attentional Blink (AB)

The AB is a rapid serial visual presentation task during which targets (e.g., letters) must be identified in a rapid stream of distractor stimuli. The attentional blink refers to the frequent failure to report a second target when it appears within close temporal proximity of a first target. It is robust, insensitive to practice effects and is thought to reflect a combination of factors including attentional control mechanisms for the selection and consolidation of targets and a suboptimal allocation of attentional resources. The AB could be described as a failure to disengage attention from the first target. Performance greatly differs between individuals. Observers with higher levels of WMC and broad attentional focus perform better than those with lower WMC and narrow attentional focus (Willems & Martens, 2016).

Nijmeijer et al. (2022) tested 53 German–English young adults who were German dominant, but diverse in a language-entropy measure. Using both (or multiple) languages in the same interactional contexts yields higher entropy scores and is often viewed as a critical active ingredient in generating beneficial effects of bilingualism. Nijmeijer et al. (2022) reported no support for the claim that language entropy, or other individual factors related to bilingual experience and use, influences the magnitude of the attentional blink (AB). Earlier studies (Colzato et al., 2008) surprisingly showed poorer attentional control, as evidenced by a larger AB in multilinguals versus monolinguals, with one exception (Kappes et al., 2023).

##### Do Tests of Attention Control Yield One-Factor Models?

**Figure 1:**
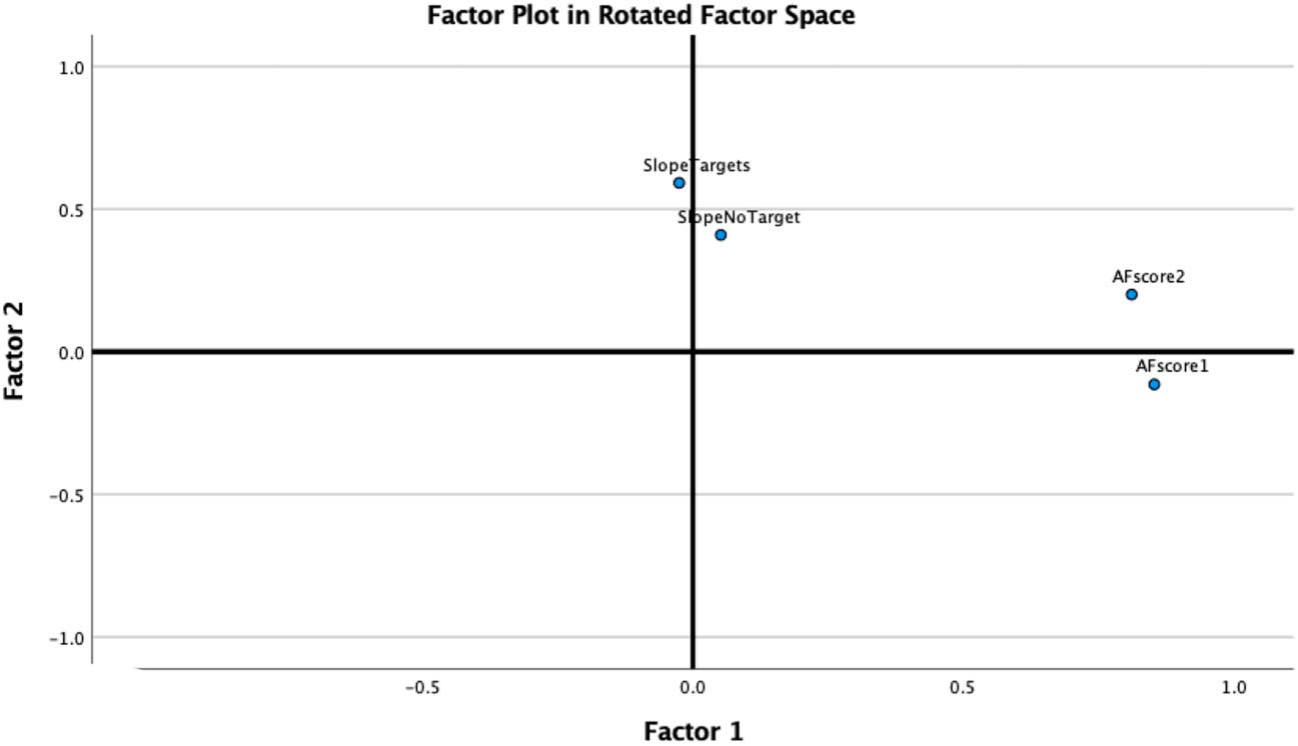
The two-factor plot of an exploratory factor analysis of the conjunctive search task (target present and target absent) and ambiguous figure task (AF1 = trial on which new stimulus no longer looks like the start object and AF2 = trial on which new object can be correctly identified).

Recall that Bialystok favors a unidimensional model of AC and eschews multidimensional models of EF like the Unity and Diversity model. If a single attention-control resource underlies the effects of bilingualism across the varied task, then participants who are good at one of the above tasks should also be good at any of the others and so forth. We can do one test of this prediction by reanalyzing the data from the 141 participants in Paap et al. (2018) to determine if the data are fit best by a one-factor or two-factor model. In this study, two measures of AC were derived from the ambiguous figures task. AF1 was the trial on which a participant indicated that the interpretation of the current drawing no longer looked like the start object. AF2 was the trial on which a participant correctly identified the new object. Similarly, two measures of AC were obtained in the conjunctive visual-search tasks as the slope of search time as a function of the number of distractors on target present trials and also on target absent trials. Both tasks are assumed to provide a sensitive measure of individual differences in the ability to disengage attention. When an exploratory factor analysis is performed on these four measures, the fit to a two-factor model is clearly superior to a one-factor model. More revealing (as shown in Figure 1 for the factor-loading plot), there is no hint that the two tasks have any common mechanism instrumental to performance.

## Part 2. Bialystok (2024) Bilingualism Modifies Cognition Through Adaptation, Not Transfer

### The Role of Transfer Versus Adaptation in the Bilingual-Advantage Debate

In her recent *Trends* article, Bialystok (2024) argues that “…bilingual experience modifies cognition through an adaptation to the underlying attention system, making attention more efficient…. In this view, bilinguals require less attentional effort than monolinguals for similar levels of performance, and outperform monolinguals on tasks with high attention demands” (p. 1). This article appears to be a reaffirmation of Bialystok and Craik’s (2022) selective-attention theory for how bilingualism modifies cognitive function. It sets up a contrast between “adaptation” and “transfer” that might explain why robust bilingual advantages are so elusive despite bilinguals having “more efficient” attention control. In this section, we briefly describe why the distinction between adaptation and transfer may be a bit of a red herring and reiterate the goal of testing theories that are falsifiable.

We quibble with Bialystok when she suggests that “The standard explanation for bilingual effects on cognition is that an aspect of language processing transfers to nonverbal cognitive performance, leading to improvements in executive functioning.” Counter to Bialystok’s assertion that transfer is central to the standard explanation for bilingual effects on cognition, its role in the bilingual advantage debate has been minimal. A search for published articles containing “bilingual advantage” returned 3,847 articles, but only 10 also contained the term “far transfer.” Thus, “far transfer” cannot be the standard expression for the standard explanation for the bilingual advantage.

The “far transfer” account discussed by Bialystok takes a narrow view of transfer much like Thorndike’s seminal theory of identical elements (Thorndike & Woodworth, 1901). Thorndike concluded that transfer occurs when two situations share common elements. The more similar the two situations, the more likely transfer will occur. The elements in Thorndike’s theory are not abstract or generalizable skills; they are concrete, specific features of the tasks that can be directly mapped from one to another. This type of identical-elements transfer would not predict, and therefore cannot account for, a positive effect of managing two languages on nonverbal cognition. Here we have a point of agreement with Bialystok (2024). Furthermore, it is probably safe to speculate that no one believes that transfer (in the narrow Thorndikian sense) can or does produce bilingual advantages in domain-general measures of EF/AC.

However, Paap (2023) did embrace a special variant of far transfer. He explicitly referred to far transfer as a possible mechanism by which bilingualism might enhance EF, but clarifies that he is describing the type of transfer that would be logically required to account for a bilingual advantage in nonverbal measures of EF: If learning to speak a second language and coordinate it with a native language produces far transfer to nonverbal tasks, then three conditions would have to be true: (1) bilingual-language control routinely recruits a domain-general attention-control mechanism, (2) this domain-general control mechanism grows stronger with use, and (3) this same domain-general control mechanism is recruited by the far-transfer task and consequently benefits from (2).

If one adopts this more expansive view of transfer, then the distinction between transfer and adaptation is a matter of emphasis*.* If learning a second language leads to enhancements in performance-based measures of attention control, it is useful to describe the research question as a test of far transfer. Alternatively, if one focuses on the brain changes that take place during L2 learning, then it seems natural to refer to these changes as adaptation. Both emphases are useful, especially in different contexts. Whether or not far transfer occurs (whether bilinguals perform better than monolinguals on nonverbal tests of EF) as a result of managing two languages is an important theoretical question and also has implications for public policy. Mapping neural changes to various aspects of bilingual experience (i.e., to adaptation) will eventually explain why performance-based transfer does or does not occur.

Currently, the effects of bilingualism on nonverbal tests of attention are small and inconsistent, and as these tests become more sophisticated, they cast more doubt that there are specific constellations of bilingual experience that reliably enhance domain-general EF. For example, Paap, Majoubi, Anders-Jefferson, et al. (2025) conducted a rigorous test of the bilingual advantage, examining the relationship between bilingualism—measured as a latent variable comprising six continuous facets of bilingual use—and attention control, represented by five performance-based measures, including Engle’s “Square” tests (Burgoyne et al., 2023). Despite the difficulty of the tasks, the relationship between the bilingualism and an AC latent variables was near zero. These findings align with most meta-analyses of the bilingual advantage and second-order meta-analyses (e.g., Gobet & Sala, 2023; Paap, Majoubi, Balakrishnan, & Anders-Jefferson, 2025), which conclude that bilingualism, like other forms of brain training, does not reliably produce far transfer.

### Managing Two Languages May Not Involve Adaptive Control

We noted that far transfer quite naturally refers to a behavioral outcome whereas adaptation has neuroscience connotations, for example, as a mechanism for experience-dependent neural plasticity. In this section, we want to consider a related but more specific construct, adaptive control, which refers to the brain’s ability to flexibly regulate attention, behavior, and cognitive resources in response to changing task demands or environmental conditions. It builds on the idea that cognitive control isn’t static – rather, it adapts based on context, performance feedback, and goals. Bialystok and Craik’s (2022) AC theory fits this general description of adaptive control and explains how bilinguals can better modulate attention based on situational cues during language production. The only necessary knock-on assumption is that ubiquitous coordination of two languages leads to a strengthening of these domain-general adaptive-control processes.

Throughout the bilingual-language debate, there have been few challenges to the intuitive assumption that adaptive control of attention is necessary for the everyday management of two languages. In contrast, Spinelli and Sulpizio (2024) reported a clever challenge to this assumption. They leveraged a well-researched method for inducing adaptive changes in the allocation of attention, viz., the proportion-congruent effect. Take a task long-associated with attention control, e.g., the Stroop color–word interference task where participants must, on incongruent trials, selectively attend to a task-relevant dimension (the color of the ink) and disengage attention from an irrelevant but salient dimension (an incongruent printed word). The incongruent trials obviously have longer RTs than the congruent trials, but the magnitude of this congruency effect depends on the proportion of incongruent trials. When incongruent trials are rare (e.g., 25%), there is less motivation to engage resource-demanding attention compared to when incongruent trials are common (e.g., 75%). In Spinelli and Sulpizio (2024), the congruency effect was 97 ms in the mostly congruent lists and only 63 ms in the mostly incongruent list. The Congruency × Proportion interaction was highly significant and shows that participants are more willing to engage in effortful proactive control when they know that such preparation will be needed more often than not. This replicates many previous studies manipulating proportion correct in tasks that require AC. Does this pattern extend to a task requiring BLC?

If, in everyday use, bilinguals need to selectively attend to the current target language, then they receive ubiquitous practice in engaging and disengaging domain-general adaptive control. This may be especially true when unbalanced bilinguals are speaking in their less proficient language. Spinelli and Sulpizio reasonably view this as the central component of BLC (i.e., a constant need to deal with irrelevant information from the nontarget language), unlike other aspects of BLC which have turned out to be somewhat epiphenomenal (e.g., language-switching effects, see Blanco-Elorrieta & Pylkkanën, 2018). This logic invites the use of an L2 picture-naming task as a diacritical test of the assumption that BLC routinely recruits and adapts attention control. In this task, Italian–English bilinguals named pictures in their L2 (English). The picture-naming task sets up a competition for selection when the translation equivalents are noncognates (casa – house). The competition is mild or absent for cognates (e.g., telefono–telephone). Consistent with this difference in competition, RTs to noncognates take longer to name than cognates.^9^ For the present purpose, the critical question is whether manipulating the proportion of cognates produces the same pattern of interaction as manipulating the proportion of incongruent trials in the Stroop task. It did not. The interaction was not significant as the cognate effect was about 87 ms in both the mostly cognate lists and the mostly noncognate lists. If domain-general AC is a hallmark of BLC, then the mostly cognate condition should have motivated proactive AC and significantly better performance, just like the mostly incongruent trials in the Stroop task.

In summary, BLC in everyday life may not require or recruit domain-general AC. It may make do with language-specific mechanisms to handle competition between a bilingual’s two languages. This possibility is discussed in Part 3’s section on skill acquisition and automaticity.

### Is the Sea of Null Results Due to the Use of Easy Tasks?

Bialystok (2025) repeats an argument presented earlier in Bialystok and Craik (2022) that attributes inconsistencies in the bilingualism literature to a key factor: the positive effects of bilingualism are most evident when attentional demands are sufficiently high. The N-back task serves as a prime example. In this task, participants view a sequence of stimuli and determine whether the current stimulus matches one presented exactly *n* steps earlier. For instance, in the sequence (1) cow–(2) pig–(3) cow, a correct response in a 2-back task would be “yes” for (3), as “cow” appeared two trials prior. Naturally, a 2-back task is more challenging than a 1-back task. Difficulty increases further with the inclusion of “lures,” stimuli that match earlier items but at incorrect intervals, e.g., (1) cow–(2) pig–(3) pig (Teubner-Rhodes et al., 2016).

Research by Janus and Bialystok (2018) with 9-year olds and Barker and Bialystok (2019) with young adults demonstrated bilingual advantages in the more demanding 2-back condition, but not in the simpler 1-back condition. These findings align with Bialystok and Craik’s (2022) graded-difficulty hypothesis. However, in both studies, bilingual participants, while more accurate, were slower—a caveat that complicates the interpretation. More compellingly, Comishen and Bialystok (2021) reported greater performance declines for monolinguals compared to bilinguals as task difficulty increased in a study involving 64 young adults. This evidence supports the idea that bilingual advantages manifest under heightened cognitive demands but are absent in simpler tasks such as 1-back.

Bialystok (2025) explains the lack of bilingual advantages observed in other studies, assuming null results arise predominantly from easy conditions. Yet this assumption is problematic, as null findings are not restricted to low-demand tasks. It is unsurprising that Bialystok’s laboratory demonstrated, on three occasions, no group differences in an easy 1-back condition for the tested age groups. The more critical question is whether other laboratories can replicate bilingual advantages in standard N-back tasks where performance is well below ceiling levels. The N-back task is often grouped with working memory capacity (WMC) span measures. Meta-analyses on WMC, encompassing diverse tasks, reveal small, nonsignificant effect sizes after correcting for publication bias. Specifically, Lehtonen et al.'s meta-analysis examined five studies using the *N*-back task and found a mean effect size of −0.29, favoring monolinguals, with a range from −0.75 to +0.17. As performance in these studies was consistently below ceiling, one might conclude that this evidence favored monolingual advantages.

Additionally, Lukasik et al.'s (2018) large-scale study compared 176 monolinguals, 115 early bilinguals, and 150 late bilinguals. Using a genetic matching algorithm, groups were equated on relevant background variables. Participants (*M*_age_: 33 years) completed a composite WMC score derived from a visuospatial WM task, a verbal WM task, and both verbal and visuospatial 2-back tasks. The 2-back tasks, featuring lures, proved challenging, with accuracy averaging around 55%. The results showed late bilinguals outperforming both monolinguals and early bilinguals, who did not differ. Given that early bilinguals are usually considered better candidates for EF advantages, these results are puzzling. Lukasic et al. suggest that the late bilingual advantage could reflect a selection bias, with individuals possessing superior inherited EF more likely to succeed in acquiring a second language when starting later than early childhood. Setting late bilinguals aside, the Lukasic et al. study provides robust evidence from a well-matched large sample, showing that early bilinguals do not consistently outperform monolinguals in demanding N-back tasks.

## Plausible Reasons for Why Bilingualism Does Not Enhance EF/AC

The meta-analyses reviewed above show that the size of the bilingual-advantage in executive function (EF) or attention control (AC) is small and near zero when corrected for publication bias. Although many are drawn to the hypothesis that bilingualism enhances domain-general EF, there are three synergistic reasons why the true effects of bilingualism on EF may be trivial at best. These reasons are developed next under the rubrics of dilution, heritability, and automaticity.

### Dilution and Ceiling Effects

Despite the small effect size in the meta-analyses, assume for now that bilingualism does enhance domain-general EF. If it does, then it is unlikely that it would be the sole environmental factor. Other often mentioned candidates include education, music, videogames, mindfulness, nutrition, and exercise.^10^ If multiple environmental factors independently enhance EF, the effect size for any single factor (e.g., bilingualism) will likely be smaller relative to overall variation in EF. Thus, even if bilingualism contributes to EF, its influence could be overshadowed by the combined effects of other factors, making the bilingual advantage harder to detect statistically. We call this scenario “dilution.”

Some of these EF-enhancing factors may be unevenly distributed between monolinguals and bilinguals. In some populations, bilinguals may have higher average exposure to education or SES which could enhance EF. Alternatively, multilinguals might face more SES challenges in other contexts which could counteract their EF advantages. As the number of potential confounders grows, it becomes harder to determine whether EF differences are truly due to bilingualism or other variables.

The status of confounding aside, the dilution effect becomes even more pronounced if the effects of environmental enhancers follow an exponential function to an asymptotic maximum. In this case, the additional contribution of bilingualism to EF may be negligible or indistinguishable, as most individuals have already reaped the bulk of potential EF benefits from other sources. This creates a saturation effect, where any marginal contribution from bilingualism is effectively drowned out. To be clear, in the asymptotic portion of the curve, individuals with many enhancers will have EF scores that cluster closer to the maximum. This leads to reduced variance among high-functioning individuals and a diminished ability to detect differences attributable to bilingualism because there is less room for bilingualism to show a measurable impact. For individuals at the lower or middle portions of the curve (where environmental enhances have stronger effects), the variability introduced by these enhancers will increase. This variability adds noise to the data, further masking the contribution of bilingualism. Detecting small or residual effects, particularly near the asymptote, requires much larger sample sizes than usually recommended to achieve sufficient statistical power.

### Heritability

To this point, the discussion has assumed that individual differences in EF have both genetic and environmental origins. This issue is related to, but not identical with, the construct of heritability. Heritability is the proportion of variance of a phenotype explained by genetic influences in a population of individuals. Phenotype refers to a characteristic that has been measured and, in this case, might be performance on a task assumed to require EF or the loading on a latent variable at the component level (e.g., switching) or to common EF. Heritability is often estimated from studies of monozygotic (MZ, identical) and dizygotic (DZ, fraternal) twins. The underlying logic is based on the fact that MZ twins share all of their genes whereas DZ twins share only about half their genes. If one further assumes that genes have additive effects and that both types of twins have the same shared environment, then several key inferences can be drawn from the correlations obtained within each type of twin. For example, the additive assumption suggests that a DZ correlation should be about half the size of the MZ correlation. If the DZ correlation is more than half the MZ correlation, then shared environment is implicated. To take another constraint, if genetic and shared-environment effects accounted for all the variance, then the MZ correlation should be 1.0. Thus, to the extent that the MZ correlation is less than 1.0 provides an index of the amount of unshared environmental influence. When these constraints are formally expressed in a structural-equation model, estimates can be derived for the variance explained by heritability, shared environment, and unshared environment.

#### Likelihood of Bilingualism as an Environmental Factor

Turning to the case at hand, we are considering the possibility that bilingualism enhances common EF. The remainder of this section explores the plausibility of this hypothesis given that common EF is not highly heritable. A provocative place to start is Friedman et al.’s (2008) large-scale twin study showing that the latent variables for both common EF (99% heritable) and componential EFs (greater than 65% heritable) are almost entirely genetic in origin. This places EFs among the most heritable psychological traits. Only the latent variable for switching shows a small (13%) significant contribution from the nonshared environment. Surprisingly, the main theme is that both the unity and diversity of EF have genetic origins. Unity, of course, because shared EF is 99% heritable. But the separability of the updating and shifting components are not due to influences from the environment, but mostly from additional genetic influences that are independent from those accounting for common EF.

One caveat to any simple interpretation of these results is that possible Gene by Environment (G-E) interactions are subsumed into the “heritability” component (unless they are explicitly included in the model). G-E interactions occur when different genotypes respond differently to the same environment. Consider an infant born into a family of balanced bilinguals and two possible G-E interactions. It could be that rich exposure to two languages benefits only those with high genetic EF. Or it could be the reverse. If either is true, then bilingualism can enhance EF, but only certain genotypes will reap the benefit. A related concept (see Plomin et al., 1977) is G-E correlation: Different phenotypes are selectively exposed to different environments. Children with high (or low) EF are likely to be treated differently by others and to personally seek compatible environments. G-E correlations are important to consider in studies of sequential bilingualism when a second language is acquired after mastery of a native language. When measures of EF and L2 proficiency positively correlate, is it because managing two languages enhances EF, or is it because having high EF makes L2 learning easy and fun?

The study by Engelhardt et al. (2015) provides a second look at the heritability question and does so in a younger population (8- to 15-year-old twins and triplets) where heritability estimates of cognitive abilities are often much lower (Haworth et al., 2010). With only 65% of the sample identifying as non-Hispanic White, and with 31% reported having received a form of mean-tested public assistance, this sample drawn from central Texas was more diverse racially and socioeconomically than the sample from Colorado tested by Friedman et al. (2008).^11^

Despite the differences in population, the results are strikingly similar at the highest level. The common EF factor was 100% heritable, indicating that correlations among the four components in their model (viz., inhibitory control, switching, working memory, and updating) are entirely attributable to shared genetics. One point of difference is that Engelhardt et al.’s study with children showed that both Working Memory and Updating were also influenced by nonshared environment. If EF is 99% heritable, only 1% of the variance in EF within the targeted population can be attributed to environmental factors. This drastically limits the potential effect size of bilingualism on EF, as bilingualism would need to compete with other environmental influences (e.g., education, nutrition, SES) for that 1%.

If the environment is homogenous, a high-degree of heritability can be misleading. However, the Engelhardt et al. (2015) study cannot be readily dismissed on these grounds.

#### Other Environmental Influences

These findings raise critical questions about the role of environmental influences on EF in general. In WEIRD (Western, Educated, Industrialized, Rich, and Democratic; Henrich, 2020) societies, many individuals are exposed to similar enriching activities, such as education, bilingualism, and extracurricular pursuits. Heritability estimates reflect the proportion of variance attributable to genetic differences within a specific population and environmental context. If cognitively demanding activities such as bilingualism, music performance, or mindfulness are common, their effects on heritability estimates depend on the variability and dynamics of their influence across the population. If these environmental factors are widespread and uniform, they contribute to baseline EF levels but do not explain individual differences. As a result, heritability estimates remain high, as they are driven by the variance between individuals rather than the absolute contribution of environmental factors. Said another way, activities such as bilingualism, music performance, or mindfulness may enhance EF capacity within individuals but only modestly increase population-level variability. This is because these activities elevate EF across the board rather than selectively enhancing it in a subset of the population. Heritability metrics, which rely on variance, would therefore attribute more of the observed differences to genetic factors, even though environmental factors play some role in shaping overall EF capacity. We are not aware of any analysis that maps out how uniform these environmental factors would have to be to generate heritability estimates of, say, 99% – perhaps extraordinarily so.

### The Role of Skill Acquisition (Automaticity) in Bilingual Language Control

Highly skilled performance does not require high levels of domain-general EF. Theories of skill acquisition have long emphasized the transition from the slow, deliberate, and conscious control in early stages of training to the fast, automatic, and unconscious control available to the expert (Chein & Schneider, 2012; Posner & Snyder, 1975). The important control mechanisms for language production and comprehension may be specialized within a language-processing module. Even if the control demands of a bilingual are intense (as, for example, in simultaneous interpretation), only the specialized mechanism would become stronger. There would be no reason to expect far transfer to a nonverbal task or to any presumed measure of domain-general EF. From this perspective, bilingual advantages—if any—might be restricted to the early stages of L2 acquisition when domain-general EF may play an important role in preventing interference from L1. This prediction is explicit in Paap’s (2018) controlled-dose hypothesis that assumes that gains in domain-general EF will not be maintained if the demands drop below a current baseline either because a frequently practiced skill has become automatic or through disuse (e.g., if a language is no longer used because of immigration or other circumstances).

In an excellent review and critical analysis, Lehtonen et al. (2023) referred to this hypothesis as the skill learning or task-specificity account. They emphasize that in everyday conversations, bilinguals decide when to switch (they are not instructed to switch by an “experimenter”) and use natural cues that are acquired through statistical learning. If natural language switching runs on automatic and relies on well-learned natural cues, then it is no mystery why good language switchers are not necessarily good task switchers when asked to switch between artificial tasks using arbitrary cues.

The core argument here is that the BLC acquired by reasonably proficient bilinguals no longer requires domain-general EF and, consequently, using two languages does not lead to a bilingual advantage in domain-general EF. If language selection and switching have become mostly automatic, this accounts for why proficient bilinguals do not invest their conscious processing on deciding if and when to shift languages. They have developed a specialized and automatic system for making these decisions that rarely needs to be corrected.

To be explicit, if bilingual language control becomes automatic, there are implications for the bilingual-advantage debate and for the heritability of EF. If proficient bilinguals do not frequently engage domain-general EF, there would be no reason to expect bilingualism to enhance it. This would predict advantages in early L2 learners that diminish or disappear in proficient bilinguals. Any advantages should be temporary: stronger when L2 is still effortful, weaker when L2 becomes automatic. Turning to heritability, if environmental effects such as bilingualism do not strongly influence EF, then the proportion of EF variance explained by genetics will be larger. This would push heritability estimates of EF closer to the upper-bound values actually found in the twin studies. Automaticity should also weaken gene-environment interactions if bilingualism mostly effects task-specific adaptations rather than domain-general EF.

## Voir La Vie En Rose: Reflections on the Bilingualism Debate

The debate over bilingualism’s impact on EF has been shaped by strong claims, but the weight of evidence suggests that any cognitive benefits are small, inconsistent, and often disappear when accounting for methodological rigor and publication bias. While Bialystok’s work has been influential in promoting the idea of a bilingual advantage, meta-analyses and large-scale studies consistently challenge this perspective. Three key factors—dilution from other cognitive enhancers, the high heritability of EF, and the automaticity of BLC – likely explain why bilingualism does not reliably enhance domain-general cognition. Rather than viewing bilingualism as a pathway to cognitive enhancement, a more nuanced approach is needed – one that recognizes its linguistic and social benefits without overstating its effects on nonverbal EF. Moving forward, future research should prioritize rigorous methodologies, better control for confounding variables, and explore alternative explanations for bilingual cognitive performance.

One of the persistent shortcomings of the bilingual advantage literature is its failure to specify a clear cognitive or neural mechanism by which bilingual experience would lead to general improvements in EF/selective attention. The assumption seems to be that mere practice or frequent use of EF-demanding processes will, by itself, lead to greater efficiency in domain-general EF. However, proponents of the bilingual-advantage hypothesis rarely articulate why or how this practice-based enhancement would occur. Would it increase the overall ‘pool’ of processing capacity? Would it reduce interference between competing neural representations, thereby ‘freeing up’ EF resources? Would it strengthen connectivity between prefrontal networks? These questions remain largely unanswered, making the bilingual advantage hypothesis theoretically underspecified.

In contrast, research on cognitive enhancement in other domains has begun to articulate testable mechanisms. For example, recent work reviewed by Paap (2024) suggests that cognitive capacity (CC) can be increased via structural and functional brain changes, such as improved neural efficiency, increased processing bandwidth, or heightened synaptic plasticity. A CC framework provides a more rigorous foundation for investigating how cognitive experiences might translate into general performance improvements. If bilingualism were to enhance EF, future research should move beyond vague claims about “practice” or “adaptation” effects and directly test whether bilingual experience leads to measurable increases in cognitive capacity through well-defined neural and cognitive pathways as outlined by Paap (2024). Without this shift to theory-based testing, the field risks continuing a cycle of ambiguous claims unsupported by strong mechanistic evidence.

Ellen Bialystok’s significant contributions to the field of bilingualism and cognition are undeniable, shaping how researchers think about bilingual advantages across multiple domains. However, critical examination of her claims reveals a need for greater nuance and rigor in interpreting findings. While Bialystok asserts that bilingual experiences yield nonverbal cognitive benefits, the evidence is far from conclusive. Meta-analyses and large-scale studies consistently challenge the notion of bilingual advantages, especially in domains such as gF and EF.

The assumption that bilingual advantages emerge primarily in high-demand tasks offers a plausible explanation for some findings but falls short of addressing the broader array of null results reported by independent laboratories. Moreover, claims regarding the mechanisms underpinning bilingual advantages—whether through selective attention, far transfer, or adaptation—require further empirical scrutiny. The growing consensus in the literature suggests that bilingualism, like other forms of cognitive training, may not produce widespread, domain-general enhancements in cognitive performance.

Ultimately, Bialystok’s reflections remind us of the dynamic nature of science: hypotheses evolve, theories adapt, and evidence accumulates to refine our understanding. While the narrative of bilingualism as a cognitive enhancer may be compelling, it must remain grounded in robust, reproducible data. As the field moves forward, it is vital to balance optimism with skepticism, ensuring that the search for cognitive benefits does not overshadow the complexities and nuances inherent in bilingual experiences. La vie n’est pas rose.

## Footnotes

^1^
A broader discussion of these two frameworks is presented in chapters 17 and 20 of Paap (2023).

^2^
In this respect, Theory Testing aligns with Bayes Factor analyses.

^3^
Many published tests for EF advantages in bilinguals compared to monolinguals routinely check and verify that the groups are not confounded by differences in gF. This behavior would make little sense if researchers believed that bilingualism routinely enhanced both EF and gF.

^4^
The one exception was a significant monolingual advantage in the Simon interference effect in Study 3 of Paap and Greenberg (2013).

^5^
See chapter 20 of Paap (2023) for a detailed summary of our BF results.

^6^
Although not explicitly stated, the selective-attention models discussed in Bialystok (2024, 2025) seems conceptually the same as the Attention-Control model introduced in Bialystok and Craik (2022). We assume that Bialystok is using a common theoretical framework for these three more-or-less contemporaneous expositions.

^7^
Bialystok does not elaborate regarding the difference in between a dimension of AC and a facet of AC, e.g., should different facets load on the same or different latent variables?

^8^
See Paap, Myuz, et al. (2019) and chapter 8 of Paap (2023) for the detailed arguments for points (b) and (c).

^9^
This is often referred to as the cognate facilitation effect, but that may be misleading given that the RT differences are most likely due to the difficulty of the noncognate trials, not some facilitation (e.g., redundancy gain) intrinsic to the cognate trials.

^10^
See Paap’s (2024) article on cognitive capacity and cognitive reserve for a more complete discussion of the role of EF enhancers and demoters across the lifespan.

^11^
An environmental factor is most likely to reduce heritability when it is evenly distributed (50–50) in the population, as this maximizes its contribution to phenotypic variance. When the distribution is highly skewed, environmental contributions are minimized, and genetic differences dominate the observed variance, resulting in higher heritability estimates.

## Notes

**Authorship:** Kenneth Paap, conceptualization, writing – original draft, writing – review & editing; John Majoubi, conceptualization; writing – review & editing. All authors approved the final version of the article.
